# The Role of Low Soil Temperature for Photosynthesis and Stomatal Conductance of Three Graminoids From Different Elevations

**DOI:** 10.3389/fpls.2019.00330

**Published:** 2019-03-18

**Authors:** Leonie Göbel, Heinz Coners, Dietrich Hertel, Sandra Willinghöfer, Christoph Leuschner

**Affiliations:** ^1^Plant Ecology, Albrecht von Haller Institute for Plant Sciences, University of Göttingen, Göttingen, Germany; ^2^Soil Science of Tropical and Subtropical Ecosystems, Büsgen Institute, University of Göttingen, Göttingen, Germany

**Keywords:** adaptation to cold soil, leaf respiration, photosynthesis, soil frost, stomatal conductance, transpiration

## Abstract

In high-elevation grasslands, plants can encounter periods with high air temperature while the soil remains cold, which may lead to a temporary mismatch in the physiological activity of leaves and roots. In a climate chamber experiment with graminoid species from three elevations (4400, 2400, and 250 m a.s.l.), we tested the hypothesis that soil temperature can influence photosynthesis and stomatal conductance independently of air temperature. Soil monoliths with swards of *Kobresia pygmaea* (high alpine), *Nardus stricta* (lower alpine), and *Deschampsia flexuosa* (upper lowland) were exposed to soil temperatures of 25, 15, 5, and -2°C and air temperatures of 20 and 10°C for examining the effect of independent soil and air temperature variation on photosynthesis, leaf dark respiration, and stomatal conductance and transpiration. Soil frost (-2°C) had a strong negative effect on gas exchange and stomatal conductance in all three species, independent of the elevation of origin. Leaf dark respiration was stimulated by soil frost in *D. flexuosa*, but not in *K. pygmaea*, which also had a lower temperature optimum of photosynthesis. Soil cooling from 15 to 5°C did not significantly reduce stomatal conductance and gas exchange in any of the species. We conclude that all three graminoids are able to maintain a relatively high root water uptake in cold, non-frozen soil, but the high-alpine *K. pygmaea* seems to be especially well adapted to warm shoot – cold root episodes, as it has a higher photosynthetic activity at 10 than 20°C air temperature and does not up-regulate leaf dark respiration upon soil freezing, as was observed in the grasses from warmer climates.

## Introduction

**FIGURE 1:**
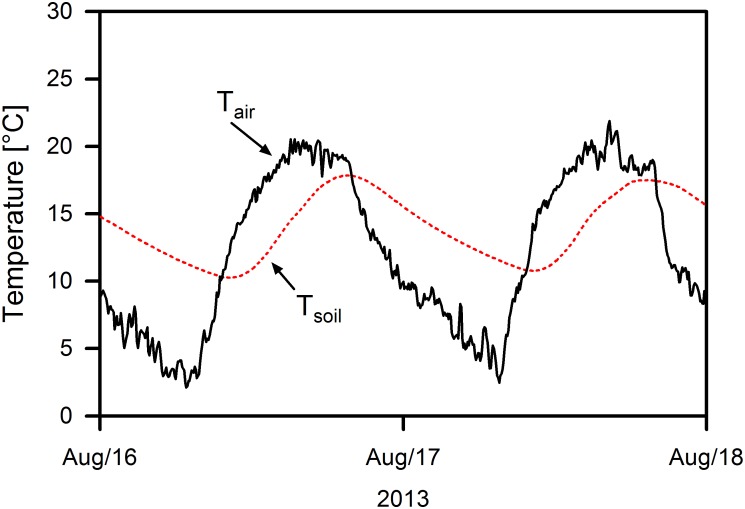
Daily fluctuation of air and soil temperature at 1 cm depth in a *Kobresia* sward at Kema Research Station (Tibetan Plateau, 4400 m a.s.l.) on 2 days in August 2013.

Plant metabolism is exposed to the thermal regime of both air and soil, which follow different diel and seasonal dynamics. Soil temperature fluctuation usually lags behind that of air temperature due to the relatively high heat capacity and low heat conductance of the soil. When the soil surface is exposed to high radiation input during the day and substantial long-wave radiation loss during the night, air and soil temperature can diverge substantially for several hours. This phenomenon of diverging soil and air temperatures occurs regularly in alpine grasslands and dwarf shrub heaths above the tree line in high mountains, where the reduced density of the air causes pronounced radiative cooling of soil and vegetation surfaces during the night under clear sky. On sunny mornings after clear nights air temperature may exceed soil temperature substantially (Figure 1). In alpine grasslands and dwarf shrub heaths, leaf surface temperature can exceed air and soil temperature by 15 K or more around noon (e.g., Körner and De Moraes, 1979). Especially in the beginning and at the end of the growing season, the shoots may then experience favorable thermal conditions for photosynthesis, while the roots are often exposed to temperatures <5°C or even face frost. Under these conditions, impairment of root growth and resource uptake activity are likely, leading to a possible mismatch in the physiological activity of aboveground and belowground organs.

There are several direct and indirect pathways by which low soil temperatures may impair plant metabolism and growth, first through reductions in root growth and decreased root water/nutrient uptake, and second indirectly through reduced photosynthetic carbon gain due to stomatal closure in the course of cold-induced drought stress (DeLucia et al., 1992; Shimono et al., 2004; Alvarez-Uria and Körner, 2007). Sachs (1875) observed that plants from warmer climates wilted when exposed to cold soil of 3–5°C, while plants from cooler climates did not. Numerous examples of plants reducing transpiration rate at soil temperatures below 10°C are given by Kramer (1969), but in most cases it remained unclear, whether physical constraints (increased viscosity of water) or physiological limitations (such as altered root membrane permeability) were the main causes of reduced water uptake. Some of these soil temperature effects on plant metabolism and growth seem to take place in partial or full independence of air temperature. An interesting question arising from these observations is whether plants exposed to warm air-cold soil conditions have developed specific adaptations to the frequent occurrence of steep aboveground-belowground temperature gradients.

Kramer (1942) formulated the hypothesis that plants from environments with frequent exposure to cold soil should develop root systems with greater hydraulic conductivity than plants evolved in habitats with warmer soil. He showed this for water melon and cotton (warm season plants) as opposed to collards (cold season plants), and for two pine species from southern and more northern parts of the United States. In contrast, Anderson and McNaughton (1973) found no clear evidence of higher stomatal conductance, photosynthesis and transpiration rates under conditions of cold soil in plants from higher elevation as compared to lower elevation in California. Possible mechanisms are alterations in root membrane structure, greater axial hydraulic conductivity, or allocation shifts toward higher root:shoot ratios under conditions of cold soil. Various studies examining the response of root growth to low soil temperature have shown that herbs, grasses, and seedlings of woody plants reduce root growth greatly below ∼5°C and cease growth at soil temperatures of 3 to 0°C (e.g., Alvarez-Uria and Körner, 2007; Gaul et al., 2008; Nagelmüller et al., 2016). There is evidence of considerable species differences with respect to root growth in cold soil (Schenker et al., 2014), but no proof was found that root growth of plants from cold climates is less sensitive to temperatures between 0 and 5°C than that of plants from warmer climates (Alvarez-Uria and Körner, 2007).

Until now, it is unclear whether low soil temperature is as influential on plant metabolism as low air temperature. Some studies have found that, under certain conditions, soil temperature may have an even larger influence on growth than air temperature, as it influences root physiology more directly. For example, Xu and Huang (2000) found high soil temperatures to be more detrimental than high air temperatures for the growth of the grass *Agrostis palustris*. The photosynthesis of *Picea rubens* saplings was more affected by soil temperature than by air temperature (Schwarz et al., 1997). Kato et al. (2005) identified soil temperature to be more important for the net ecosystem exchange of CO_2_ of grasslands on the Tibetan Plateau than air temperature.

Here, we present the results of a climate chamber experiment, in which we compared the leaf gas exchange (photosynthesis, leaf dark respiration, and transpiration) and stomatal conductance of water vapor of three graminoid species (two Poaceae and one Cyperaceae) from largely different elevations (high alpine, lower alpine and lowland) and growing season temperatures while being exposed to different soil (-2–25°C) and air temperatures (10 and 20°C). With respect to soil temperature, we distinguished between chilling temperatures (defined here as 0–10°C) and sub-zero temperatures. The studied populations belong to common and widespread Eurasian calcifuge grassland species with relatively low productivity, which differ in their elevational distribution range. As we were interested in possible inheritable adaptations of the root system to cold soil, we collected swards of the three species with attached intact root system at elevations of 250, 2200, and 4400 m a.s.l. by soil coring. The swards with the original soil were kept for several months in the greenhouse in Göttingen to overcome assumed initial stress responses of the plants to the altered temperature and light regime in the lowlands. Thus, we investigated possible inheritable adaptations of the three populations to the local thermal regime they had experienced at 250, 2200, and 4400 m a.s.l. elevation in their life. The soil and air temperature manipulation experiments lasted only for about 14 h per treatment in order to investigate only short-term temperature effects and not longer-term thermal acclimation. This enabled the plants to adjust physiologically to the altered temperatures, but excluded any possible root morphological or anatomical modifications. In order to assess the physiological importance of steep soil-to-air temperature gradients for alpine grassland plants, we tested the hypotheses that (1) soil temperature affects leaf gas exchange independently of air temperature, and (2) the species from highest elevation responds in its leaf gas exchange less sensitively to cold soil than the species from warmer environments. This study provides much needed information on how a non-uniform thermal environment influences plant physiology. Since most physiological experiments are conducted in climate chambers under more or less homogeneous thermal conditions, some of the obtained results are difficult to extrapolate to field conditions, in particular in alpine environments with steep temperature gradients.

## Materials and Methods

### Plant Material

The experiments were conducted with three graminoid species from the temperate and submeridional zones in Europe and Central Asia, which differ in the elevation of their main occurrence (Table 1). We chose species from the Poaceae and Cyperaceae families with similar growth habit (low-growing perennial tussock grasses with needle-shaped leaves), which have a distribution center either in the lowlands, in the lower alpine belt, or the high alpine belt in order to cover a broad altitudinal range. *Deschampsia flexuosa* (L.) Trin. was selected as a lowland taxon; it occurs in European grasslands and the herb layer of forests from the lowlands to the alpine belt (up to 2270 m a.s.l.) and rarely achieves dominance in the communities in which it is present. *Nardus stricta* L. (Poaceae) was chosen as a typical alpine grass species, which is present in European pastures from the lowlands to the alpine belt (up to 2600 m a.s.l.), but rarely penetrates the forest. The chosen high alpine sedge *Kobresia pygmaea* (C.B. Clarke) (Cyperaceae) dominates major parts of the Tibetan Plateau at elevations of 4000–5000 m a.s.l., the largest alpine ecosystem on earth with an extension of c. 450 000 km^2^. Hereafter, the species will be referred to by their genus names. In the summer months, the average daily temperature amplitude ranged from 6.5 to 12.6 (Table 1).

**Table 1:** Origin of the plant material used in the experiments.

Species	Origin	Altitude (m a.s.l.)	Collection date	Site characterization	Temperature at origin^1^ (°C)	Minimum temperature at origin^2^ (°C)	Maximum temperature at origin^2^ (°C)	Daily temperature amplitude^3^
*Kobresia pygmaea*	Kema Research Station, Nacqu, Tibetan Plateau31°16′N, 92°06′E	4410	Sept 2012/ Sept 2013	High-alpine pasture on acid soil	8.3	0.2–3.4	12.9–15.7	12.6
*Nardus stricta*	Visperterminen, Valais, Switzerland46°15′N, 7°56′E	2209	Oct 2012	Alpine pasture on acid soil	6.7	-0.3–5.8	6.3–12.6	6.5
*Deschampsia flexuosa*	Ebergötzen, central Germany51°34′N, 10°05’E	244	Oct/Nov 2013	Clearing of a Norway spruce plantation with medium light intensity	12.0	2.5–11.3	12.5–21.2	10.1


*Climate data: 1960–1990, source: WorldClim (http://www.worldclim.org). ^*1*^Growing season mean: Apr-Oct (Ebergötzen), May-Sept (Visperterminen), June-Sept (Kema; shorter period, since no plant growth before monsoon-onset in June). ^*2*^Mean minimum and maximum temperate at origin during growing season. ^*3*^Mean diurnal range (mean of monthly difference between daily maximum and minimum during growing season).*

In the three grasslands, soil monoliths with intact swards on top were excavated by pushing plexiglass cylinders of 15 cm diameter and 20 cm length into the soil. The cylinders were transported to the Experimental Botanical Garden of Göttingen University (51°33′N 9°57′E, 181 m a.s.l.), where the swards were kept at 15°C (daytime) and 13°C (nighttime) (12 h day length) at 110–130 μmol photons m^-2^s^-1^ in a glasshouse (metal halid lamps: EYE Clean-Ace MT400DL/BH, Iwasaki-Electric CO., LTD., Tokyo, Japan). To ensure optimal drainage, a smaller plexiglass tube filled with fiber wicks was installed at the bottom of each cylinder (Coners et al., 2016). For removing older brown leaves and promoting the regrowth of young leaves, the swards were cut to a uniform length c. 1 cm above the soil surface in case of *Kobresia* and *Nardus*, and to c. 3 cm in case of *Deschampsia*. The gas exchange measurements were conducted with the freshly regrown leaves, which were not older than 3 months. To avoid nutrient limitation during the experiments, plants were fertilized with a customary NPK-fertilizer (Wuxal, Manna, Düsseldorf, Germany) every 3 weeks and, if necessary, were treated with the biological insecticide Spruzit Neu (Neudorff, Emmerthal, Germany) against aphids. Plants were regularly watered in response to the water consumption of the swards for maintaining relatively uniform soil moisture levels in all monoliths throughout the experimental period.

### Study Design

The experiments were conducted in two fully climatized walk-in growth chambers. Two sets of experiments were conducted, the first one with an air temperature (*T*_a_) of 20°C, the second one with the same plant material and the same setup 7 weeks later with *T*_a_ = 10°C. For the measurement campaign at *T*_a_ = 10°C, 1 day before starting the measurements, plants were moved to the second plant growth chamber (Johnson Controls, Milwaukee, WI, United States) which allowed to maintain lower temperatures. Between the two measurement sets, plants were kept 7 weeks at an intermediate air temperature of 15/13°C (daytime/nighttime), assuming that a possible acclimation to 20°C air temperature had disappeared. During the 12 h-daylight period in both chambers, radiation intensity was 110–130 μmol photons m^-2^s^-1^ and relative air humidity was 75%, corresponding to a vapor pressure deficit (vpd) of 17.5 hPa at *T*_a_ = 20°C and of 12.8 hPa at *T*_a_ = 10°C. We decided to keep relative air humidity constant and not vpd, as this is more realistic than a constant vpd. During nighttime, air temperature was reduced to 15 and 8°C, respectively. Soil temperature (*T*_s_) was manipulated in the monoliths independently from air temperature by establishing each three different *T*_s_ levels at 20°C air temperature (5, 15, and 25°C) and at 10°C air temperature (-2, 5, and 15°C).

The soil monoliths were placed in a randomized block design with six replicates per species and soil temperature treatment, i.e., 6 × 3 = 18 monoliths per species (54 monoliths in total). Each block consisted of three monoliths per species, which were exposed to different soil temperatures at a given air temperature level, resulting in nine monoliths per block.

**FIGURE 2:**
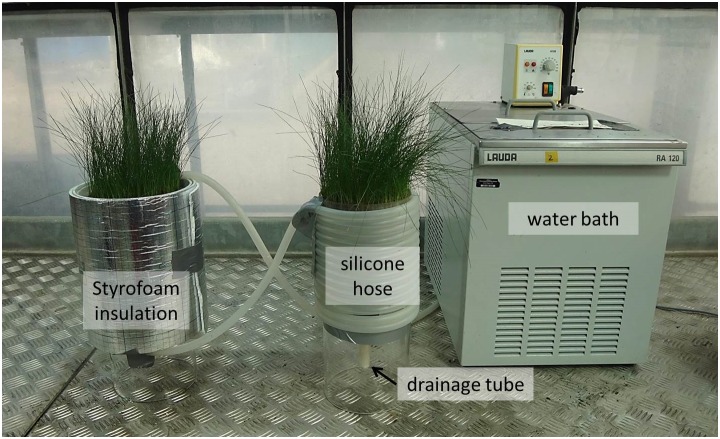
Measurement setup. A water bath is cycling heated or cooled water through hoses wound tightly around each soil monolith with grass swards on top. The styrofoam insulation buffers against external temperature fluctuations.

To achieve the desired soil temperature, water was heated or cooled in a thermostatic water bath (RM 6 or RA 120, Lauda GmbH, Lauda-Königshofen, Germany) and cycled through a silicone hose that was wound tightly around each cylinder (Figure 2). To minimize water temperature fluctuation within the system, monoliths and connecting hoses were insulated with 2 cm customary Styrofoam. In order to avoid freezing in the water bath, a customary antifreeze mixture was added to the system for the -2 and 5°C treatments.

During the experiments, soil temperature was monitored in each monolith with two miniature temperature sensors (Thermochron DS1922L-F5#, Maxim integrated, San Jose, United States) inserted to 3 cm soil depth at the center of each monolith and logged continuously. To ensure that the desired soil temperatures were reached, *T*_s_ was measured additionally with mobile thermometers (DET 3R, Voltcraft, Wollerau, Switzerland) in the swards immediately before the beginning of a measurement. Measured soil temperatures deviated from the target values by 0.1–3.0 K, with higher temperatures typically occurring during the first hours after switching on the light; in general, larger temperature deviations occurred at 20 than at 10°C air temperature (Table 2).

**Table 2:** Means (±standard error) of measured soil temperatures (in °C) in the four soil temperature treatments (-2, 5, 15, and 25°C) established in growth chambers with either 10 or 20°C air temperature.

Target soil temperature	Air temperature level	
	10°C	20°C
-2°C	-2.3 ±-0.1	-
5°C	5.1 ± 0.0	8.0 ± 0.1
15°C	15.2 ± 0.0	16.3 ± 0.0
25°C	-	26.3 ± 0.1

### Leaf Gas Exchange Measurements

After an equilibration period of at least 12 h to a target soil temperature and additional 2 h of acclimation to the full light conditions in the chamber, leaf gas exchange parameters were recorded with a portable IRGA system (Li-6400, Licor, Lincoln, Nebraska, United States). Gas exchange measurements were conducted first under ambient light intensity (∼110 μmol photons m^-2^s^-1^; A_amb_), afterward at light saturation of photosynthesis (1500 μmol photons m^-2^s^-1^; A_max_), and finally in the dark (leaf dark respiration; *R*_d_). Leaves were allowed to acclimatize to the given light intensity for 5 min in every step. Leaf temperature was adjusted to air temperature, atmospheric CO_2_ concentration was set to 400 ppm, relative air humidity to 53%.

As it was impossible to place the needle-shaped graminoid leaves in a single non-overlapping layer into the measuring chamber of 6 cm^2^ aperture, 20–30 living, green leaves each were spread in a single layer without foliage overlap and fixed with adhesive tape on a thin plate of opaque plastic, which contained a circular hole in the center (area 1.54 cm^2^). The plastic plate was then placed into the plexiglass chamber of the Li-6400 system. Since only the hole was illuminated by the artificial light source, photosynthetic activity should have largely been restricted to the 1.54 cm^2^ in the hole, while leaf dark respiration from leaf sections beyond the hole may also have been recorded. We thus assume that our net photosynthesis figures underestimate carbon dioxide assimilation to a certain extent. However, the error likely was similar in all measurements.

Stomatal conductance was measured independently of the Li-6400 measurements with a porometer (AP4, Delta-T Devices, Cambridge, United Kingdom). In a similar manner as for the Li-6400 measurements, a defined number of leaves were placed adjacent to each other to cover the cuvette aperture completely. We averaged over each three measurements of stomatal conductance per monolith and treatment.

### Statistical Analyses

All data were tested for normality with a Shapiro-Wilk test. As the assumption of normality was not met, the data were transformed to ranks and all subsequent analyses performed with the transformed data. To test for significant soil temperature effects, mixed effects models were performed separately for the two air temperature treatments with species and soil temperature as fixed effects (*p* ≤ 0.05). Additional mixed effects models with the fixed factors soil, air temperature and species were run for the soil temperature treatments *T*_s_ = 5 and 15°C and the air temperature treatments *T*_a_ = 10 and 20°C (temperature difference 10 K for both variables) in order to test for the importance of air and soil temperature on gas exchange parameters.

Pairwise Kruskal-Wallis tests were used to test for significant differences between the treatments of a species and significant differences between species for a treatment (significance level: *p* ≤ 0.05). If necessary, a Bonferroni correction was applied to account for multiple testing. All statistical analyses were performed with the software SAS (version 9.4, SAS Institute, Cary, NC, United States).

## Results

The mixed model analysis conducted separately for the two air temperature levels (10 and 20°C) showed that soil temperature had a significant effect on light-saturated net photosynthesis, leaf dark respiration, leaf conductance and transpiration rate only in the 10°C air temperature treatment (Table 3). This effect disappeared at 20°C air temperature (except for stomatal conductance, *p* < 0.05, *F* = 3.32). Mixed models including both air and soil temperature and species revealed the dominant effects of air temperature and species on leaf gas exchange, while the soil temperature effect was insignificant in all species in these overall models (Table 4).

**Table 3:** Results of mixed model analyses for light-saturated net photosynthesis, leaf dark respiration, stomatal conductance, and transpiration rate at light saturation conducted separately for the two air temperature levels (10 and 20°C) testing for the factors soil temperature, species, and the interaction of both.

Effect	Df	F-value	*p*-value
**10°C air temperature**			
**Photosynthesis at light saturation**			
Soil temperature	2	44.41	<**0.0001**
Species	2	7.49	**0.002**
Soil temperature^∗^species	4	0.44	0.78
**Leaf dark respiration**			
Soil temperature	2	5.20	**0.009**
Species	2	5.88	**0.005**
Soil temperature^∗^species	4	1.07	0.38
**Stomatal conductance**			
Soil temperature	2	64.17	**<0.0001**
Species	2	12.64	**<0.0001**
Soil temperature^∗^species	4	1.54	0.21
**Transpiration at light saturation**			
Soil temperature	2	13.58	**<0.0001**
Species	2	23.99	**<0.0001**
Soil temperature^∗^species	4	1.76	0.15
**20°C air temperature**						
**Photosynthesis at light saturation**			
Soil temperature	2	0.92	0.41
Species	2	59.72	**<0.0001**
Soil temperature^∗^species	4	0.32	0.87
**Leaf dark respiration**			
Soil temperature	2	1.13	0.33
Species	2	2.37	0.11
Soil temperature^∗^species	4	1.18	0.33
**Stomatal conductance**			
Soil temperature	2	3.32	**<0.05**
Species	2	36.09	**<0.0001**
Soil temperature^∗^species	4	0.98	0.43
**Transpiration at light saturation**			
Soil temperature	2	0.01	0.99
Species	2	19.15	**<0.0001**
Soil temperature^∗^species	4	0.51	0.73


*Significant factors (*p* ≤ 0.05) are printed in bold.*

**Table 4:** Results of mixed model analyses for light-saturated net photosynthesis, leaf dark respiration, stomatal conductance, and transpiration rate at light saturation for two soil (5 and 15°C) and air temperature treatments (10 and 20°C) with 10 K temperature difference testing for the factors soil temperature, air temperature, species, and their interactions.

Effect	Df	F-value	*p*-value
**Photosynthesis at light-saturation**			
Soil temperature	1	0.94	0.34
Air temperature	1	6.86	**0.01**
Species	2	33.35	**<0.0001**
Soil temperature^∗^air temperature	1	0.07	0.80
Soil temperature^∗^species	2	0.05	0.95
Air temperature^∗^species	2	4.21	**0.02**
Soil temperature^∗^air temperature^∗^species	2	0.60	0.55
**Leaf dark respiration**			
Soil temperature	1	1.33	0.25
Air temperature	1	21.07	**<0.0001**
Species	2	3.38	**0.04**
Soil temperature^∗^air temperature	1	0.58	0.45
Soil temperature^∗^species	2	2.80	0.07
Air temperature^∗^species	2	0.24	0.78
Soil temperature^∗^air temperature^∗^species	2	0.23	0.80
**Stomatal conductance**			
Soil temperature	1	2.64	0.11
Air temperature	1	45.64	**<0.0001**
Species	2	13.85	**<0.0001**
Soil temperature^∗^air temperature	1	0.47	0.49
Soil temperature^∗^species	2	1.55	0.22
Air temperature^∗^species	2	5.41	**0.01**
Soil temperature^∗^air temperature^∗^species	2	0.06	0.94
**Transpiration at light-saturation**			
Soil temperature	1	0.16	0.69
Air temperature	1	34.23	**<0.0001**
Species	2	21.33	**<0.0001**
Soil temperature^∗^air temperature	1	0.01	0.91
Soil temperature^∗^species	2	0.15	0.86
Air temperature^∗^species	2	4.30	**0.02**
Soil temperature^∗^air temperature^∗^species	2	0.12	0.88


*Significant factors (*p* < 0.05) are printed in bold.*

**FIGURE 3:**
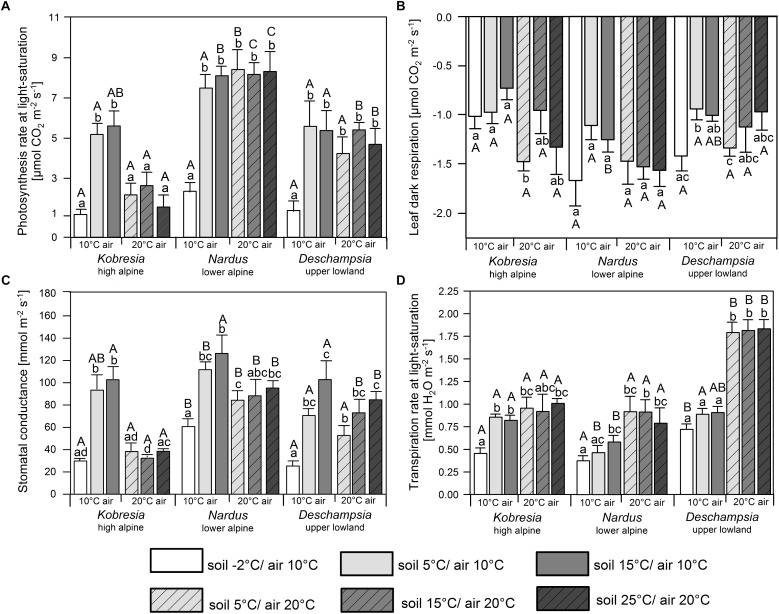
**(A)** Photosynthesis at light saturation (1500 μmol photons m^-2^s^-1^), **(B)** leaf dark respiration, **(C)** stomatal conductance, and **(D)** transpiration at light saturation of *Kobresia pygmaea*, *Deschampsia flexuosa*, and *Nardus stricta* in the two air temperature (10 and 20°C) and four soil temperature (–2, 5, 15, and 25°C) treatments. Small letters indicate significant differences (*p* ≤ 0.05) between treatments for one species. Capital letters indicate significant differences (*p* ≤ 0.05) between species within a treatment. Error bars indicate standard errors, *n* = 6.

Soil temperature shifts from 25 to 15 and 5°C had no significant effect on A_max_ at 20°C air temperature in any of the species. Warmer air temperatures had a negative effect on the photosynthesis of *Kobresia*, i.e., A_max_ was more than twice as high at 10°C air temperature than at 20°C, when soil temperature was ≥5°C (Figure 3A). In contrast, the photosynthetic activity of *Nardus* and *Deschampsia* was not affected by a *T*_a_ decrease from 20 to 10°C, when the soil was not frozen. As a consequence, *Kobresia* had a much lower A_max_ at 20°C than the two Poaceae, but had a similar photosynthetic activity as *Deschampsia* at 10°C, while the photosynthetic activity of *Nardus* was higher at any air temperature. Soil freezing (-2°C) at 10°C air temperature resulted in very low A_max_ rates (1.1–2.3 μmol m^-2^s^-1^) in all three species (Figure 3A).

Leaf dark respiration was relatively uniform across the temperature treatments and species (mostly 1.0–1.5 μmol m^-2^s^-1^) with only few significant differences (Figure 3B). In *Nardus* and *Deschampsia*, a non-significant tendency toward somewhat higher *R*_d_ rates at 20 than 10°C air temperature was visible. More striking were the high *R*_d_ rates in frozen soil (-2°C) at 10°C air temperature in these two species; *Kobresia* reached average *R*_d_ rates of 1.0 μmol m^-2^s^-1^ at a soil temperature of -2°C.

Stomatal conductance was strongly reduced in all species at -2°C soil temperature (Figure 3C). At 10°C air temperature, reductions in comparison to the warmer soil treatments were up to 70% in *Kobresia* (to 30 mmol m^-2^s^-1^), 50% in *Nardus* (61 mmol m^-2^s^-1^), and 75% in *Deschampsia* (25 mmol m^-2^s^-1^). Remarkably, all three species showed higher stomatal conductance rates at 10°C air temperature than 20°C in the 5 and 15°C soil temperature treatments (significant differences in *Kobresia*, only tendencies in *Deschampsia* and *Nardus*).

The temperature response of transpiration deviated from that of photosynthesis. Transpiration rates at 20°C air temperature were much higher in *Deschampsia* than in *Nardus* and *Kobresia* (*p* ≤ 0.05), while at 10°C *Deschampsia* and *Kobresia* transpired at similar rates, and more than *Nardus*, unless the soil was frozen (Figure 3D). In frozen soil (-2°C) *Kobresia* and *Nardus* exhibited lower transpiration rates (∼0.5 mmol H_2_O m^-2^s^-1^) than *Deschampsia* (∼0.75 mmol m^-2^s^-1^).

## Discussion

### Chilling Sensitivity of Photosynthesis and Stomatal Conductance

In our study with three graminoids from a broad range of elevations, the soil temperature effect on photosynthesis and transpiration was in all species weak at soil temperatures ≥5°C, even though the investigated temperature range in the experiments was large (5–25°C). A positive effect of higher *T*_s_ on gas exchange rates was only noticed for stomatal conductance in *Deschampsia*, which increased between 5 and 25°C (differences only partly significant, Figure 3C). This disproves our hypothesis (1) on a general air temperature-independent effect of soil temperature on gas exchange. Our climate chamber results also do not confirm the assumption that adaptation to low soil temperatures is physiologically more important than adaptation to low air temperature, as has been proposed by e.g., Schwarz et al. (1997). Similar results were obtained by Anderson and McNaughton (1973) who found neither decreases in net photosynthesis nor transpiration rate for 12 herb and dwarf shrub species, when soil temperature was reduced from 20 to 3°C. A few of the 57 plant species investigated by Döring (1935) also showed no reduction in water transport, when exposed to low root temperatures close to the freezing point, but the majority did. Several other authors reported reductions in water uptake or transpiration and photosynthesis at low soil temperatures in tropical or subtropical and temperate species (e.g., Kramer, 1942; Böhning and Lusanandana, 1952; Cox and Boersma, 1967). Part of the discrepancy in results on the soil temperature influence may be due to different experimental conditions, i.e., plants in soil vs. hydroponics and plants taken from the wild vs. plants grown in the glasshouse. Only few studies have been conducted with graminoids, to which our results can be compared. For example, rice plants reared in hydroponics reduced their transpiration rate at rhizosphere temperatures of 13°C compared to 25°C (Kuwagata et al., 2012). Similarly, Shimono et al. (2004) found a reduced stomatal conductance of rice in cold rooting medium. These results may point at a particularly high soil temperature sensitivity of graminoids from warm environments. The C4 grass *Andropogon gerardii* reduced photosynthesis greatly when grown at soil temperatures of 5 and 10°C compared to 15–40°C (DeLucia et al., 1992), which likely was caused by water stress due to root chilling. It appears that chilling effects on the photosynthesis and stomatal conductance of graminoids do occur, but seem not to be omnipresent in plants from cool environments.

That reduction in soil temperature from 25 to 5°C (at 20°C air temperature), or from 15 to 5°C (at *T*_a_ = 10°C), did not significantly decrease transpiration rate in our experiments, is at first sight surprising, as root water uptake apparently was not impaired by the substantially higher viscosity of water in cold soil. The viscosity of water at 15°C is about 33%, and at 5°C even c. 70% higher than at 25°C. This hinders water movement not only in the rhizosphere, but also in the root apoplasm and the xylem conduits of root and shoot. A possible explanation of our findings is that aquaporin activity in root membranes was higher in the colder treatments, or alternatively, the driving soil-to-leaf water potential gradient was larger, possibly due to lowered leaf water potential. In the absence of data on aquaporin activity and leaf water status, this must however remain speculative.

Leaf dark respiration differed in our experiments only little between the three species under a given thermal regime, which contrasts with photosynthesis and leaf conductance that revealed greater species differences. This shows that graminoids from cold and warm climates, when grown at the same temperature, converge in their leaf respiratory activity and maintain similar rates, even though they must have developed adaptations to largely different thermal regimes. In addition, the species from lower elevations (*D. flexuosa* and *N. stricta*) were in general not more responsive to soil temperatures <10°C, i.e., the corresponding change in gas exchange was not larger, than in the high-alpine species *K. pygmaea*. Adaptation to warmer environments thus did not lead to greater sensitivity of stomatal conductance and photosynthesis to cold soil, and the elevation of plant origin did not influence the response in our sample of graminoid species. While *K. pygmaea* is distributed only above ∼3000 m a.s.l. (Flora of China, 2015), *N. stricta* is abundant in both the lowlands and in the alpine belt to >2000 m, and *D. flexuosa* is most widespread in the lowlands and lower mountain ranges. The results from this study thus seem not supportive of the hypothesis of Kramer (1942) who predicted that species native to cooler climates should possess root systems with greater water permeability at low soil temperatures than plants native to warmer climates. However, the three species of our study are not sufficient to test this hypothesis.

### What Is Different in High-Alpine Kobresia?

The findings suggest that *Kobresia* has not only a low temperature optimum of photosynthesis, but also that the species apparently is largely insensitive to soil cooling between 15 and 5°C.

Our gas exchange data suggest that the A_max_ of *Kobresia* was reduced at 20°C due to both, a higher dark respiration rate of the leaves, and a greatly reduced stomatal conductance compared to 10°C air temperature. Since soil moisture was kept at favorable, non-limiting levels in the monoliths throughout the experiments, and cold soil cannot play a role, it was possibly stomatal sensitivity to a higher vpd that caused partial stomatal closure in *Kobresia* at 20°C air temperature. This interpretation would be in line with the observation that soil temperatures between 5 and 25°C had no influence on stomatal conductance, photosynthesis and transpiration in this high alpine species. The sedge *Carex curvula* in the alpine grasslands of the high Alps has a temperature optimum of photosynthesis of 15°C at 2300 m a.s.l., when radiation was low (200 μmol m^-2^s^-1^), but a much higher optimum of 20–25°C at 1500 μmol m^-2^s^-1^ (Körner, 1982). Since our plants were grown at relatively low light intensities in the chambers (110–130 μmol m^-2^s^-1^) in order to be able to reduce soil temperatures to low values in the chamber, it is likely that the photosynthetic apparatus of the experimental plants was adapted to these light conditions, which may relate to the low temperature optimum of A_max_. During rainless weather, insolation can be much higher on the Tibetan Plateau, suggesting that the temperature optimum of photosynthesis in *Kobresia* may also be higher in the field than observed in our experiment. In order to assess the relevance of the observed adaptive responses to cold soil in *Kobresia* under natural conditions, additional gas exchange measurements in the field during days with large air-to-soil temperature gradients are needed.

### Freezing Sensitivity of Gas Exchange

In contrast to chilling effects, soil freezing (-2°C) had a pronounced effect on the stomatal conductance, photosynthesis and (in *Kobresia*) transpiration rate of all three graminoid species in a similar manner as it is observed for conifers (Havranek and Tranquillini, 1995; Schaberg et al., 1995). The reduction in transpiration upon soil cooling from +5 to -2°C can well be explained by stomatal closure, probably caused by leaf dehydration due to impaired water uptake. However, stomatal closure explains only part of the concomitant reduction in photosynthesis. In *Nardus* and *Deschampsia*, an increase in leaf respiration has contributed to the reduction in net photosynthesis rate, but not so in *Kobresia*. This suggests that non-stomatal mechanisms such as decreased Calvin cycle enzyme activity or electron transport (Öquist et al., 1983; Teskey et al., 1995; Schwarz et al., 1997) may have played a role in this high-alpine species.

Freezing temperatures in the soil reduced photosynthesis of the three species, but not leaf dark respiration. In fact, *R*_d_ was equally high, or even higher, at -2°C than at 5 or 15°C soil temperature, suggesting that stomata closed due to impaired water supply from the soil, which affected photosynthesis but not respiration. Mitochondrial respiration in the leaves apparently was up-regulated in *Nardus* and *Deschampsia* in response to the stress imposed by soil freezing. This could be a direct response to leaf water status deterioration, or caused by a hypothetical chemical signal from the frost-exposed roots.

## Conclusion

Contrary to expectation, we could not detect an air temperature-independent effect of cool, non-frozen soil on the photosynthesis and stomatal conductance of the three graminoids from variable altitudes (hypothesis 1). This suggests that periods with warm shoot but cold root system especially at high elevations in the morning hours may allow a relatively high photosynthetic activity despite cold soil. The physiological mechanisms which allow these graminoids to maintain a relatively high root water uptake at a soil temperature of 5°C still need further investigation.

Our data further suggest that the high-alpine sedge *K. pygmaea* is especially well adapted in its gas exchange to microclimatic conditions with warm shoots and cold roots. While the other two species from warmer climates also seem to be able to cope quite well with cold soil, they apparently lack some adaptations of *Kobresia* (hypothesis 2). The sedge differed from the other species in that it did not up-regulate leaf dark respiration in frozen soil. This may indicate that *Nardus* and *Deschampsia* are developing a negative C balance during soil freezing earlier than *Kobresia*.

Our results reveal only inheritable adaptations in leaf gas exchange to low soil and air temperatures, since we conducted an experiment under uniform climatic conditions, in which the plants were grown for several months under lowland conditions prior to measurement. Moreover, the results of this experimental study can hardly be extrapolated to the field, as a species’ ability to cope with soil and air temperature variation will certainly be greater under natural conditions. Short-term reversible responses due to phenotypic plasticity will add to inherited adaptations, which were not studied here.

## Data Availability

All datasets generated for this study are included in the manuscript and/or the supplementary files.

## Author Contributions

LG conducted the climate chamber experiments and did most of the data analysis and manuscript preparation. HC, DH, and SW retrieved the sward monoliths from the field and developed and supervised the experimental design. CL had the initial idea and wrote major parts of the manuscript.

## Conflict of Interest Statement

The authors declare that the research was conducted in the absence of any commercial or financial relationships that could be construed as a potential conflict of interest.
